# Correlation between intervertebral disc degeneration, paraspinal muscle atrophy, and lumbar facet joints degeneration in patients with lumbar disc herniation

**DOI:** 10.1186/s12891-017-1522-4

**Published:** 2017-04-20

**Authors:** Dong Sun, Peng Liu, Jie Cheng, Zikun Ma, Jingpei Liu, Tingzheng Qin

**Affiliations:** Department of Orthopedics, China–Japan Union Hospital of Jilin University, 126 XianTai Street, Changchun, 130000 China

**Keywords:** Lumbar disc herniation, Disc degeneration, Multifidus muscle atrophy, Facet joints degeneration, Lumbar extension muscle strengthening program

## Abstract

**Background:**

To assess the correlation between lumbar disc degeneration (LDD), multifidus muscle atrophy (LMA), and facet joints degeneration in patients with L4-L5 lumbar disc herniation (LDH).

**Methods:**

Sixty patients with L4-L5 LDH diagnosed by a 1.5 T MRI scanner were enrolled in the study group and another 60 patients with non-specific back pain were enrolled in the control group. LDD, LMA, and facet joints degeneration were examined and analyzed independently by two independent orthopedic surgeons using T2-weighted images. Wilcoxon test was used for analyzing the difference of LDD and facet joints degeneration between L3-L4 and L5-S1 and difference of LMA between the herniated and control groups. Correlation analysis of the three degeneration grades at the same level was determined by Spearman rank correlation test.

**Results:**

In the herniated group, most LMA at L3-L4 level was grade 1 (42, 70.0%); grade 2 (33, 55.0%) at L4-L5 level; and grade 3 (27, 45.0%) at L5-S1 level. LMA and LDD grading were significantly different between L3-L4 and L5-S1 levels (*P* < 0.05). In the herniation group, the Spearman value for LDD and LMA grading were 0.352 (*P* < 0.01) at L3-L4 and 0.036 (*P* > 0.05) at the L5-S1 level. The differences in LMA between the herniated and control groups at the three levels were significant (*P* < 0.05).

**Conclusions:**

Disc degeneration and multifidus muscles atrophy were positively correlated at the L3-L4 disc level. A lumbar extension muscle strengthening program could be helpful in preventing muscle atrophy and lumbar spinal degeneration.

## Background

Lumbar disc herniation (LDH) is one of the most frequent causes of low back pain and sciatic pain in adults. The compression by the protruding disc on the dorsal and/or the ventral nerve roots causes low back pain, leg pain (sciatica), muscle spasm, and restriction of trunk movement [1]. The multifidus muscle—the most medially located back muscle and the largest muscle that spans the lumbosacral junction—serves to maintain the erector posture of the trunk and to abduct and rotate the trunk. It is also innervated by the dorsal root of the lumbar spinal nerve [2–4].

Several recent studies have investigated the biomechanics and microstructures of the multifidus muscles in patients with LDH [5, 6]. An association between multifidus muscle degeneration and chronic low back pain, degenerative disc disease, radiculopathy, and scoliosis has been proposed [7–9]. Muscle atrophy and fatty infiltration to the multifidus muscle following minimally invasive lumbar discectomy was also observed [10]. However, there have only been a few reports on magnetic resonance imaging (MRI) analysis of the paraspinal muscles [11], facet joints, and lumbar disc degeneration (LDD) in LDH patients, let alone the correlation study of lumbar degenerative changes between herniated and adjacent segments in patients with L4-L5 disc herniation. We hypothesized that lumbar multifidus atrophy (LMA) is correlated with LDH and that severe atrophy exists in the level innervated by the dorsal root of the herniation compression nerve root.

The present study aimed to compare (by MRI scanning) the differences in LMA, LDD, and facet joints degeneration between the L3-L4 and L5-S1 levels in patients with L4-L5 LDH, and to analyze the correlation between LMA and LDD at the L3-L4 and L5-S1 levels.

## Methods

### Study participants

Between January 2015 and April 2015, 60 patients who had unilateral radiculopathy due to L4-L5 level disc herniation diagnosed by 1.5 T MRI scanner as well as 60 patients (as the control group) with non-specific back pain at our university hospital were enrolled in our study. Patient characteristics are summarized in Table 1.

**Table 1:** Patient characteristics of the disc herniation group and the control group

Variables	Disc herniation group	Control group	*P* value
Age	48.97 ± 8.39	46.08 ± 7.27	>0.05
Sex, female	29 (48.3%)	31 (51.7%)	–
History (months)	9.15 ± 5.51	–	–
VAS	6.60 ± 1.33	–	–

Data is presented as mean ± standard deviation

*VAS* visual analogue scale/Score

The inclusion criteria were: no obvious disc degeneration signs such as disc collapse, endplate Modic and Schmorl changes, and high-intensity zone within the posterior annulus; confirmed L5 nerve root compression by herniated mass during surgery [5]; and aged 20 to 70 years old.

The exclusion criteria were: MRI showed L3-L4 and L5-S1 segments disc herniation; lumbar spinal canal stenosis; and history of lumbar surgery, neoplasm, infection, or spinal deformity, such as spondylolisthesis (>3 mm) or scoliosis (>10°).

### Imaging parameters

MRI of lumbar spine was taken by a 1.5 T MRI scanner (MAGNETOM® Verio, A Tim + Dot System; Siemens, Erlangen, Germany), with the participants in the supine position. And the following scans were performed using the following parameters: sagittal T2-weighted images from T12 to the sacrum (TR/TE 2980/122.6, matrix size 208 × 320, time to recovery: 3,000–3,600 ms, time to echo: 87–114 ms, and slice thickness: 4 mm); and axial T2-weighted images from T12 to S1 (TR/TE 2980/122.6, matrix size 208 × 320, time to recovery: 3,000–3,600 ms, time to echo: 87–114 ms, and slice thickness: 4 mm).

### Muscle morphometry

**Fig. 1:**
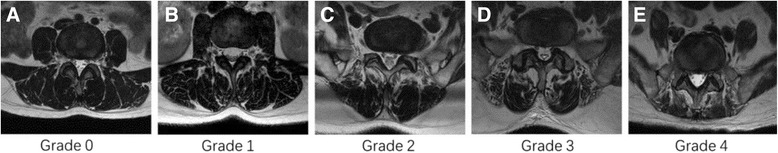
Goutallier grading (range, 0 to 4) on T1W axial MRIs are represented by (**a**) to (**e**), respectively [9, 12], and are graded as: grade 0, normal muscle tissue; grade 1, fat streaks; grade 2, more muscle than fat; grade 3, equal amounts of fat and muscle tissue; and grade 4, more fat than muscle

Multifidus muscle cross-sectional area (CSA) was determined at the levels of the L3-L4, L4-L5, and L5-S1 intervertebral discs. Multifidus CSA was measured by the semiquantitative grade system published by Patrick [12] (Fig. 1).

### Grading of intervertebral disc degeneration

The modified system comprises of 8 grades for lumbar disc degeneration [13]. The eight grades represented a progression from normal disc to severe disc degeneration. Grade 1 corresponds to no disc degeneration while Grade 8 corresponds to end-stage degeneration.

### Grading of the lumbar facet joints osteoarthritis

**Fig. 2:**
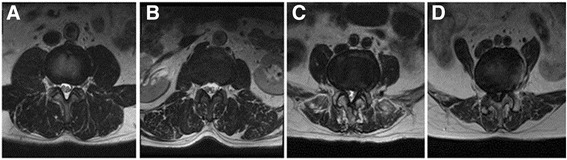
Examples of MRIs on T2W axial with Pathria grades 0 to 3 are shown [14]. Four grades of osteoarthritis of the facet joints were defined as: (**a**) grade 0, normal; (**b**) grade 1, mild degenerative disease; grade 2, moderate degenerative disease; and (**c**) grade 3, severe degenerative disease

Four grades of osteoarthritis of the facet joints were defined using system published by Pathria [14]: grade 0, normal; grade 1, mild degenerative disease; grade 2, moderate degenerative disease; and grade 3, severe degenerative disease (Fig. 2).

### Image assessment

Sagittal T2-weighted images were used to analyze disc degeneration as they provided a comprehensive perception of disc structure and good tissue differentiation, and axial T2-weighted images were used to analyze facet joints and paraspinal muscle. Two experienced orthopedic surgeons each graded 360 lumbar levels independently.

### Statistical analyses

The analyses were performed using SPSS software (version 20.0; SPSS IBM; Armonk, NY). Intraobserver reproducibility of analysis results of intervertebral disc, facet joints, and muscle CSA degeneration was assessed using weighted Kappa statistics. Wilcoxon rank sum test was used to compare the differences in intervertebral disc, facet joints, and muscle CSA degeneration grade between L3-L4 and L5-S1 as well as the differences in muscle atrophy between the herniated and control group. Spearman’s rho testing was used to analyze the correlation between LDD and LMA at L3-L4 and L5-S1.

## Results

A total of 120 patients were included in this retrospective study. There were 29 females and 31 males in the herniation group with an average age of 48.97 ± 8.39 years (range from 32 to 65 years). There were 31 females and 29 males in the control group, with an average age of 46.08 years (range from 30 to 64 years). There was no significant difference between the two groups (Table 1).

**Fig. 3:**
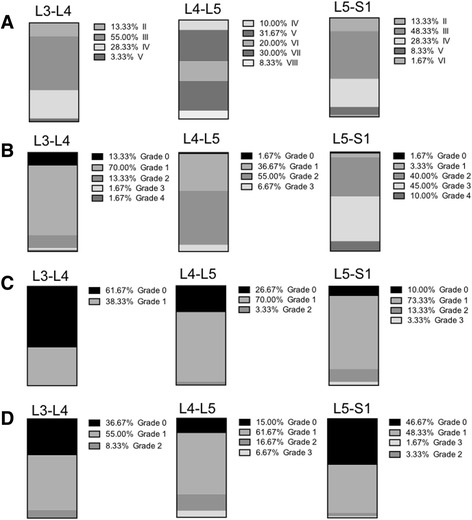
Lumbar multifidus atrophy, lumbar disc, and facet joints degeneration in the herniated and control groups. **a** lumbar disc degeneration in the herniated group; **b** lumbar multifidus atrophy in the herniated group; **c** lumbar multifidus atrophy in the control group; **d** facet joint degeneration in the herniated group

The disc degeneration grading of all patients according to the modified Pfirrmann grading system was shown in Fig. 3. Disc degeneration at the L3-L4 level disc was mostly grade III (33, 55.0%) and grade IV (17, 28.3%). L4-L5 disc degeneration was mainly grade V (19, 31.67%), grade VI (12, 20.0%), and grade VII (18, 30.0%). L5-S1 disc degeneration was grade III (29, 48.33%) and grade IV (17, 28.33%).

**Fig. 4:**
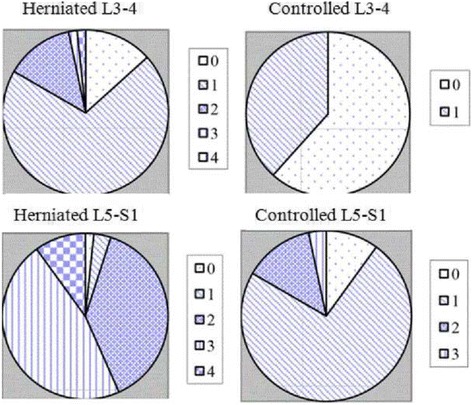
Distribution of multifidus atrophy in the herniated and control groups. 0: grade 0 of multifidus; 1: grade 1 of multifidus; 2: grade 2 of multifidus; 3: grade 3 of multifidus; 4: grade 4 of multifidus

As illustrated in Figs. 3 and 4, according to the Goutallier grading, LMA from the herniated group at the L3-L4 level was mostly grade 1 (42, 70.0%). LMA at the L4-L5 level was mainly grade 1 (22, 36.67%) and grade 2 (33, 55.0%). L5-S1 level was grade 2 (24, 40.0%) and grade 3 (27, 45.0%). As for the control group, LMA at the L3-L4 level was grade 0 (37, 61.67%) and grade 1 (23, 38.33%). LMA at L4-L5 and L5-S1 levels were mainly grade 1 (42, 70.0 and 44, 73.33%). There were significant differences in LMA at the three levels between the herniated and control group (Table 2).

**Table 2:** Wilcoxon rank sum test between herniated and control group lumbar multifidus atrophy

	L3-L4 (control)	L4-L5 (control)	L5-S1 (control)
L3-L4 (herniated)	−5.126^**^ (*P* < 0.01)		
L4-L5 (herniated)		−5.818^**^(*P* < 0.01)	
L5-S1 (herniated)			−6.217^**^(*P* < 0. 01)

**P < 0.01

The correlation between LDD and LMA at L3-L4, L4-L5, and L5-S1 levels is shown in Table 3. Significant correlation was observed at the L3-L4 level (Spearman’s rho value = 0.352, *P* < 0.05) between LDD and LMA, but no correlation was observed at the L4-L5 and L5-S1 levels.

**Table 3:** Correlation analysis of lumbar multifidus atrophy and disc degeneration by Spearman’s rho test

	L3-L4 LDD	L3-L4 LMA	L4-L5 LDD	L4-L5 LMA	L5-S1 LDD	L5-S1 LMA
L3-L4 LDD	1.000	0.352**	0.125	0.133	0.283*	0.124
L3-L4 LMA		1.000	0.243	0.591**	0.009	0.439**
L4-L5 LDD			1.000	0.058	0.097	0.006
L4-L5 LMA				1.000	−0.094	0.872**
L5-S1 LDD					1.000	−0.036
L5-S1 LMA						1.000

*LMA* lumbar multifidus atrophy, *LDD* lumbar disc degeneration

*P < 0.05; **P < 0.01

The Wilcoxon rank sum test result is shown in Table 4. A significant difference in LMA was observed between L3-L4 and L5-S1 levels (Z = - 6.481, *P <* 0.05), but no significant difference was found in other levels in LDD and facet joints degeneration (Table 4).

**Table 4:** Wilcoxon rank sum test between L3-4 and L5-S1 lumbar disc degeneration changes

	L3-L4 LMA	L3-L4 facet joints	L3-L4 LDD
L5-S1 LMA	−6.481^**^(*P* < 0.01)	–	–
L5-S1 facet joints	–	−1.143(*P* = 0.253)	–
L5-S1 LDD	–	–	−1.119(*P* = 0.263)

*LMA* lumbar multifidus atrophy, *LDD* lumbar disc degeneration

**P < 0.01

Facet joints degeneration at the L3-L4 level was mainly grade 0 and grade 1 (36.7 and 55.0%). L4-L5 level facet joints degeneration was mainly grade 1 (61.67%). Facet joints degeneration at level L5-S1 was mainly grade 0 and 1 (46.67 and 48.33%) (Fig. 3). The inter-examiner reliability analysis of LDD, LMA, and facets joints degeneration showed excellent result (Kappa = 0.91, 0.84, 0.86, and 0.85, respectively), thus indicating that the measurements were reliable.

## Discussion

In this study, we investigated the correlation between multifidus muscle atrophy, facet joints, and disc degeneration in LDH patients. The result of this study supported our hypothesis that in patients with L4-L5 LDH, multifidus muscle grading was higher at the L5-S1 level than at the L3-L4 level. Positive correlation was identified between LDD and LMA at the L3-L4 level, but no correlation was found at the L5-S1 level.

We found that compared with the control group, herniated patients had more severe LMA at the three levels. There were two possible explanations. First, it could be because the selected patients in the control group had no obvious disc degeneration symptoms. Second, multifidus muscle disuse caused by specific back pain and denervation caused by nerve root compression were the main reasons responsible for this phenomenon.

MRI results from this study demonstrated that there was more severe LMA at the L5-S1 level than at the L3-L4 level in L4-L5 LDH patients. Anatomically [15, 16], multifidus fascicles arise from the spinous process and adjacent lamina of each lumbar vertebra, descend caudolaterally, traverse L3-L4 vertebral levels, and is innervated by the nerve root of the same level. Similar to the L5 level, multifidus muscle arising from the L5 spinous process is innervated by the L5 nerve root. In patients with L4-L5 LDH, if the L5 root was affected, muscle denervation would be expected only at the L5 level. The anatomical features result in more muscle volume at the inferior segment than the superior segment.

Two mechanisms for muscle atrophy have been proposed: disuse and denervation. In animal studies [17, 18], it is generally agreed that denervation leads to decrease in Type II fiber size. In contrast, opinion differs concerning changes in the Type I fiber size after denervation. However, Yoshihara et al. [19] demonstrated that denervation caused by nerve root compression may lead to atrophy of both fiber types (type I and type II) with structural changes in the human lumbar multifidus muscle.

Despite these controversies, we proposed two mechanisms that are possibly responsible for multifidus atrophy. In LDH patients, disuse/immobilization of the back muscles is common. Such changes may explain the generalized effect (atrophy) at five levels. In addition, symptoms including paraspinal denervation (short-angled fibers) and re-innervation (grouped fibers, polyphasic action potentials) are common in disc herniation and nerve root compression. The CSA of multifidus muscle innervated by medial branch of the dorsal ramus of the L5 nerve root are reduced when L5 nerve root is compressed by herniated mass.

Numerous studies have reported rapid muscle atrophy in response to inflammation [20, 21] and muscle or joint injury [19]. However, the mechanisms for such changes are poorly understood. Paraspinal muscle plays a more important role in protecting the L3-L4 segment than others. The multifidus muscle maintains the lumbar lordosis by acting like a bowstring which helps to transmit some of the axial compression force on the disc to the anterior longitudinal ligament by switching compression loading to stretch loading; additionally, it protects the discs by preventing unexpected movements like torsion and flexion [22]. This phenomenon not only requires sufficient strength muscle, but also completes the nucleus pulposus and annulus fibrosus structure. For the L5-S1 segment, however, the intervertebral disc below the iliac crest, the ilium could restrict segment movement, thus reduce the pressure and shear forces on the intervertebral disc.

We found no correlation between L5-S1 level disc degeneration and multifidus muscle atrophy in our study. At the L3-L4 level, as there was no denervation phenomenon, the positive correlation demonstrated that LMA could be the cause of disc degeneration. At the L5-S1 level, however, the differences of LMA between the two groups suggested that LMA could also be the consequence of L4-L5 disc herniation.

This study analyzed the lumbar degenerative changes between herniated and adjacent segments in L4-L5 herniated disc patients using MRI. The multifidus muscle plays a critical role in controlling and stabilizing the lumbar spine in multiple planes of action, including axial rotation as well as posterior rotation (extension) on the sagittal plane [23].

Lower back pain can recollapse more often in case of weakened paraspinal muscles resulting in instability; the latter can be improved by rehabilitation exercise programs [24, 25]. For disc-herniated patients, microdiscectomy results in minimal atrophy and fatty infiltration to the multifidus muscle [26–29]. And lumbar extension muscle-strengthening program can decrease paraspinal muscle atrophy after lumbar disc herniation surgery, and prevent adjacent level degeneration and lumbar spondylolisthesis, especially for patients with severe LMA caused by lower lumbar range of motion (ROM) after posterior lumbar interbody fusion.

Choi et al. [30] demonstrated that pain, duration of recuperation, and back-muscle strength can be affected favorably by lumbar extension muscle-strengthening programs after lumbar disc herniation surgery. Thus, it is recommended that MRI should be performed to obtain a description of LMA in disc-herniated patients, and clinicians should pay attention to LMA when instituting lumbar extension muscle strengthening programs.

There are several limitations with this study. First, we analyzed only patients with mono-segment disc herniation at L4-L5; excluding L3-L4/L5-S1 disc herniation and multi-segment degeneration due to the relatively small sample size. Second, we were not able to perform a comparative analysis to investigate the difference between the herniated cohort and control groups. Further study with a larger number of cases should be performed to obtain an age- and sex-matched control result and thus confirm the results of the present study.

## Conclusions

This study analyzed the lumbar degenerative changes between herniated and adjacent segments in L4-L5 disc herniated patients. There was severe LMA in the L5-S1 segment. Our data confirmed that LMA contributes to the etiology of disc degeneration and/or disc herniation, especially at the L3-L4 level.

Muscle atrophy should be considered in the treatment of lumbar disc herniation patients due to its contribution to clinical symptoms. Lumbar extension muscle strengthening programs could be effective in preventing muscle atrophy and lumbar spinal degeneration.

## Abbreviations

CSA: Cross-sectional area
LDD: Lumbar disc degeneration
LDH: Lumbar disc herniation
LMA: Lumbar multifidus atrophy
MRI: Magnetic resonance imaging
ROM: Range of motion
